# Impact of hyperglycemia on myocardial ischemia–reperfusion susceptibility and ischemic preconditioning in hearts from rats with type 2 diabetes

**DOI:** 10.1186/s12933-019-0872-7

**Published:** 2019-05-31

**Authors:** Steen Buus Kristiansen, Kim Bolther Pælestik, Jacob Johnsen, Nichlas Riise Jespersen, Kasper Pryds, Marie Vognstoft Hjortbak, Rebekka Vibjerg Jensen, Hans Erik Bøtker

**Affiliations:** Department of Cardiology, Aarhus University Hospital, Skejby Sygehus, Palle Juul-Jensens Boulevard 99, 8200 Aarhus N, Denmark

**Keywords:** Type 2 diabetes mellitus, Ischemia, Reperfusion, Infarction, Hyperglycemia, O-GlcNAc

## Abstract

**Background:**

The mechanisms underlying increased mortality in patients with diabetes and admission hyperglycemia after an acute coronary syndrome may involve reduced capacity for cardioprotection. We investigated the impact of hyperglycemia on exogenously activated cardioprotection by ischemic preconditioning (IPC) in hearts from rats with type 2 diabetes mellitus (T2DM) that were endogenously cardioprotected by an inherent mechanism, and the involvement of myocardial glucose uptake (MGU) and myocardial O-linked β-*N*-acetylglucosamine (O-GlcNAc).

**Methods and results:**

In isolated, perfused rat hearts subjected to ischemia–reperfusion, infarct size (IS) was overall larger during hyper- ([Glucose] = 22 mmol/L]) than normoglycemia ([Glucose] = 11 mmol/L]) (p < 0.001). IS was smaller in 12-week old Zucker diabetic fatty rats with recent onset T2DM (fa/fa) than in rats without T2DM (fa/+) (n = 8 in each group) both during hyperglycemia (p < 0.05) and normoglycemia (p < 0.05). IPC (2 × 5 min cycles) reduced IS during normo- (p < 0.01 for both groups) but not during hyperglycemia independently of the presence of T2DM. During hyperglycemia, an intensified IPC stimulus (4 × 5 min cycles) reduced IS only in hearts from animals with T2DM (p < 0.05). IPC increased MGU and O-GlcNAc levels during reperfusion in animals with and without T2DM at normoglycemia (MGU: p < 0.05, O-GlcNAc: p < 0.01 for both groups) but not during hyperglycemia. Intensified IPC at hyperglycemia increased MGU (p < 0.05) and O-GlcNAc levels (p < 0.05) only in hearts from animals with T2DM.

**Conclusion:**

While the effect of IPC is reduced during hyperglycemia in rats without T2DM, endogenous cardioprotection in animals with T2DM is not influenced by hyperglycemia and the capacity for exogenous cardioprotection by IPC is preserved. MGU and O-GlcNAc levels are increased by exogenously induced cardioprotection by IPC but not by endogenous cardioprotection in animals with T2DM reflecting different underlying mechanisms by exogenous and endogenous cardioprotection.

## Background

Glycometabolic status at hospital admission is an independent prognostic marker of all-cause mortality in patients with acute coronary syndrome (ACS), even without a previous history of T2DM [1, 2]. The mechanisms underlying the association between hyperglycemia and increased mortality in patients with ACS are multifactorial. Release of inflammatory factors and increased platelet aggregation has been proposed [3, 4]. However, altered myocardial susceptibility to ischemia–reperfusion (IR) injury or reduced effect of cardioprotection may also be involved. Cardioprotection may be activated by inherent chronic conditions (endogenously), as observed in hearts from animals with T2DM that have an inherent cardioprotection at onset of T2DM [5–7], or activated exogenously with immediate onset by ischemic preconditioning (IPC) [8]. Recently, we reported that circulating glucose concentration influences myocardial susceptibility to ischemia–reperfusion injury and that the cardioprotective capacity by endogenous cardioprotection in animals with T2DM and by exogenously induced cardioprotection in animals without T2DM is lost during hypoglycemia [9]. Previous studies indicate that hyperglycemia increases myocardial susceptibility to ischemia–reperfusion injury and attenuates the efficacy of IPC [10, 11]. However, these studies were performed in models of type 1 diabetes and non-diabetic animals while patients with T2DM prevail in clinical patient cohorts of acute myocardial infarction.

The impact of circulating glucose concentrations on myocardial susceptibility to ischemia–reperfusion injury and the capacity for cardioprotection may be linked at a mechanistic level to myocardial glucose metabolism because cardioprotection by IPC is associated with changes in myocardial glucose uptake (MGU) during reperfusion at normoglycemia [9, 12, 13]. Since hyperglycemia may impact MGU during reperfusion, the efficacy of cardioprotection may concurrently be modified. At a molecular level myocardial glucose metabolism is linked to O-linked β-*N*-acetylglucosamine (O-GlcNAc) glycosylation, which is a posttranslational modification of proteins associated with cellular stress and death [14]. O-GlcNAc glycocylation not only seems to be dependent on circulating glucose concentrations, but also seems to be associated with cardioprotection by IPC [15, 16]. UDP-GlcNAc is the monosaccharide donor for O-GlcNAcylation and the end product of the hexosamine biosynthetic pathway (HBP), which is sensitive to alterations in circulating glucose and glutamine concentrations [17]. HBP seems to be involved in the diabetic pathophysiology and hyperglycemia may increase flux through the HBP and promote protein O-GlcNAcylation [18]. We hypothesized that hyperglycemia influences the capacity for endogenous and exogenous cardioprotection differently and myocardial susceptibility to ischemia–reperfusion injury through simultaneous changes in MGU and O-GlcNAc levels during reperfusion. We aimed to investigate the impact of T2DM and the cardioprotective effect of IPC and the concurrent changes in MGU and O-GlcNAc concentrations in an animal model of T2DM and non-diabetes during hyperglycemia.

## Methods

### Animals

Zucker diabetic fatty (ZDF) rats (homozygote (fa/fa)) and their age-matched lean controls (fa/+) at the age of 12 weeks (Charles River Laboratories, Kisslegg, Germany) were housed at 23 °C, a 12 h light/dark cycles, 30% humidity, and allowed free access to food (Purina 5008 diet) and water. We used the ZDF rat because it is a widely used and well characterized animal model of T2DM with an inherent metabolic disarray including insulin resistance, hyperinsulinemia, abnormal glucose metabolism and mitochondrial dysfunction, that may impact cardioprotection [19–22]. The study conformed to Danish law for animal research (Act no. 1306 of 23/11/2007, Danish ministry of Justice) and the Guide for the Care and Use of Laboratory Animals published by the National Institute of Health.

### Protocols

Before the experiments a tail blood was sampled after 12 h fasting to validate development of T2DM by analyzing circulating blood glucose (OneTouch^®^ Ultra Blood Glucose, lifescan Inc., CA, USA) and insulin levels (AlphaLISA^®^ Insulin Kit, PerkinElmer, MA, USA). The rats were randomly allocated to 10 experimental groups (n = 8): (I–IV) Perfusion with 11 mmol/L Glucose (normoglycemia) (I: DM Control, II: DM IPC, III: Non-DM Control, IV: Non-DM IPC), and (V–X) perfusion with 22 mmol/L Glucose (hyperglycemia) (V: DM Control, VI: DM IPC, VII: DM intensified IPC, VIII: Non-DM Control, IX: Non-DM IPC, X: Non-DM intensified-IPC). Hyperglycemia at a glucose concentration of 22 mmol/L was used as this concentration has previously been used in studies of hyperglycemia [23]. After 20 min of stabilization IPC hearts were preconditioned by 2 × 5 min of global ischemia, a stimulus that is stronger than we have previously used [5], because it has been demonstrated that an enhanced stimulus is needed to elicit cardioprotection in diabetic rats [7]. Each period of global ischemia was followed by 5 min of reperfusion. The intensified IPC protocol consisted of 4 × 5 min ischemia and reperfusion. Subsequently all hearts were subjected to 40 min of global ischemia and 2 h of reperfusion.

### Isolated perfused heart

An isolated perfused rat heart preparation was used as previously described [24, 25]. Briefly, after induction of anesthesia the rats were connected to a ventilation apparatus and the hearts were dissected from surrounding structures through a thoracotomy. The hearts were cannulated in situ and transferred to a Langendorff perfusion apparatus (IH-SR type 844/1; Hugo Sachs Electronik, Harvard Apparatus, March-Hugstetten, Germany) using a constant perfusion pressure of 80 mmHg at 37 °C and perfused with Krebs–Henseleit buffer (NaCl_2_ 118.5 mmol/L, KCl 4.7 mmol/L, NaHCO_3_ 25.0 mmol/L, glucosemonohydrate 11.0 or 22.0 mmol/L, MgSO_4_·7H_2_O 1.2 mmol/L, CaCl_2_ 2.4 mmol/L and KH_2_PO_4_ 1.2 mmol/L) at pH 7.4 and oxygenated with 5% CO_2_ and 95% O_2_. Normoglycemia at a glucose concentration of 11 mmol/L was used because this is not considered hyperglycemic in the absence of free fatty acids and other substrates in the experimental model because normal postprandial glucose concentration in rat is up to 10.4 mmol/L [26] and this is the most commonly used concentration in the Langendorff perfused heart model [27]. A fluid-filled balloon was placed in the left ventricle. Coronary flow was measured using an in-line flow probe (Type 2.5SB, Transonic Systems Inc., Ithaca, NY, USA). Hemodynamic data were acquired and analyzed using dedicated software (Notocord Hem-v3.5, Croissy sur Seine, France).

### Myocardial infarct size

After reperfusion hearts were frozen at − 80 °C for 15 min, sliced (≈ 1.5 mm), and stained with 1% 2,3,5-triphenyltetrazolium chloride, while an apical slice was immediately snap frozen in liquid nitrogen and stored at − 80 °C as previously described [28]. Area-at-risk (AAR) and area-of-infarction were assessed by computer planimetry (UTHSCA ImageTool, San Antonio, TX, USA). Infarct size (IS) was subsequently calculated as area-of-infarction/AAR and weighted with the weight of each slice weight. All measurements were done blinded.

### Myocardial glucose uptake rate

MGU was assessed from rates of ^3^H_2_O production from D-[2-^3^H]-glucose [29]. Tracer perfusion was used during stabilization and 0–30 min of reperfusion. Samples were collected as a baseline arterial sample and multiple effluent samples and stored at − 80 °C until analyzed. Glucose uptake rate was calculated as previously described [6].

### O-GlcNAc western blot

Heart slice was snap-frozen in liquid nitrogen immediately after end of reperfusion. Samples were thawed and homogenized in ice-cold extraction buffer with added enzyme activity inhibitors (PIC2, PIC3, KF, B-glycerophosphate, TSA 1, Thiamet-G and PMSF). Protein concentration of the supernatant was determined using Pierce 660 nm Protein Assay (Thermo Scientific). Western blotting was performed by priming with antibodies: anti-O-GlcNAc antibody (CDT 110.6, Gift–CoreC4) and anti-actin antibody (Sigma). Densitometry was calculated relatively to densitometry of the total protein blot bands and control-band of each blot. Results were presented as relative to non-DM control rats perfused with glucose concentration of 11 mmol/L.

### Statistical analysis

Analyses were blinded. Glucose uptake, western blotting densitometry, stabilization hemodynamics, blood-insulin and -glucose level, and rat weights were analyzed using one-way ANOVA with post-test when appropriate. Infarct size was compared using one-way ANOVA and specific comparisons between groups were performed with *t*-test or one-way ANOVA with post-test when appropriate. Hemodynamics during reperfusion was presented as left ventricular developed pressure (LVDP) (LVDP = LV systolic pressure − LV diastolic pressure) and rate pressure product (RPP) (RPP = LVDP × heart rate) and compared using two-way ANOVA with repeated measurements. Two-sided p-value < 0.05 was considered significant. Values are presented as mean ± SEM.

## Results

### Animals

Fasting blood glucose (11.3 ± 0.7 mmol/L vs. 5.2 ± 0.2 mmol/L, p < 0.001), insulin concentration (5.7 ± 1.4 μIU/mL vs. 0.6 ± 0.3 μIU/mL, p < 0.001) and bodyweight (356 ± 8 g vs. 291 ± 4 g, p < 0.001 was higher in animals with than without T2DM. Heart weight/body weight was smaller in animals with than without diabetes (p < 0.01), while heart weight did not differ between groups (p = 0.11).

### Infarct size

**Fig. 1:**
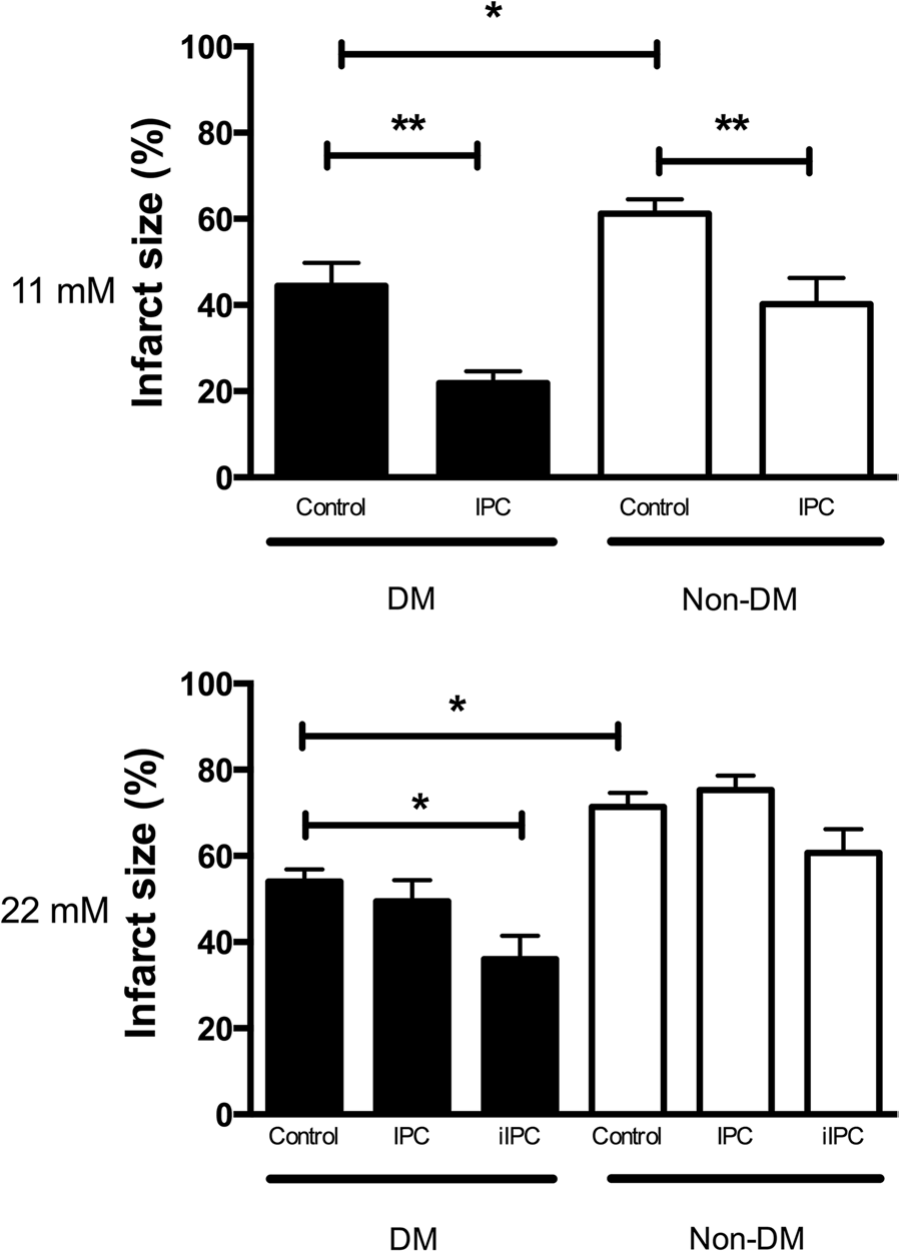
Infarct size (%) at the end of 40 min of global ischemia and 120 min of reperfusion in diabetic (DM) Control, Non-DM Control, DM ischemic preconditioned (IPC) and Non-DM-IPC hearts. Perfusion glucose level were 11 mmol/L and 22 mmol/L. *p < 0.05; **p < 0.01. Mean ± SEM

Overall, myocardial infarct sizes were larger during hyperglycemia than during normoglycemia in rats with and without T2DM (p < 0.001). Infarct size was smaller in animals with than without T2DM during normo- (p < 0.05) and hyperglycemia (p < 0.05) (Fig. 1). IPC reduced myocardial infarct size in animals with (p < 0.01) and without (p < 0.01) T2DM during normoglycemia but not at hyperglycemia. Intensified-IPC reduced myocardial infarct size during hyperglycemia in animals with T2DM (p < 0.05) but not in animals without T2DM (p = 0.46). Area-at-risk did not differ between groups (p = 0.21).

### Hemodynamics

**Fig. 2:**
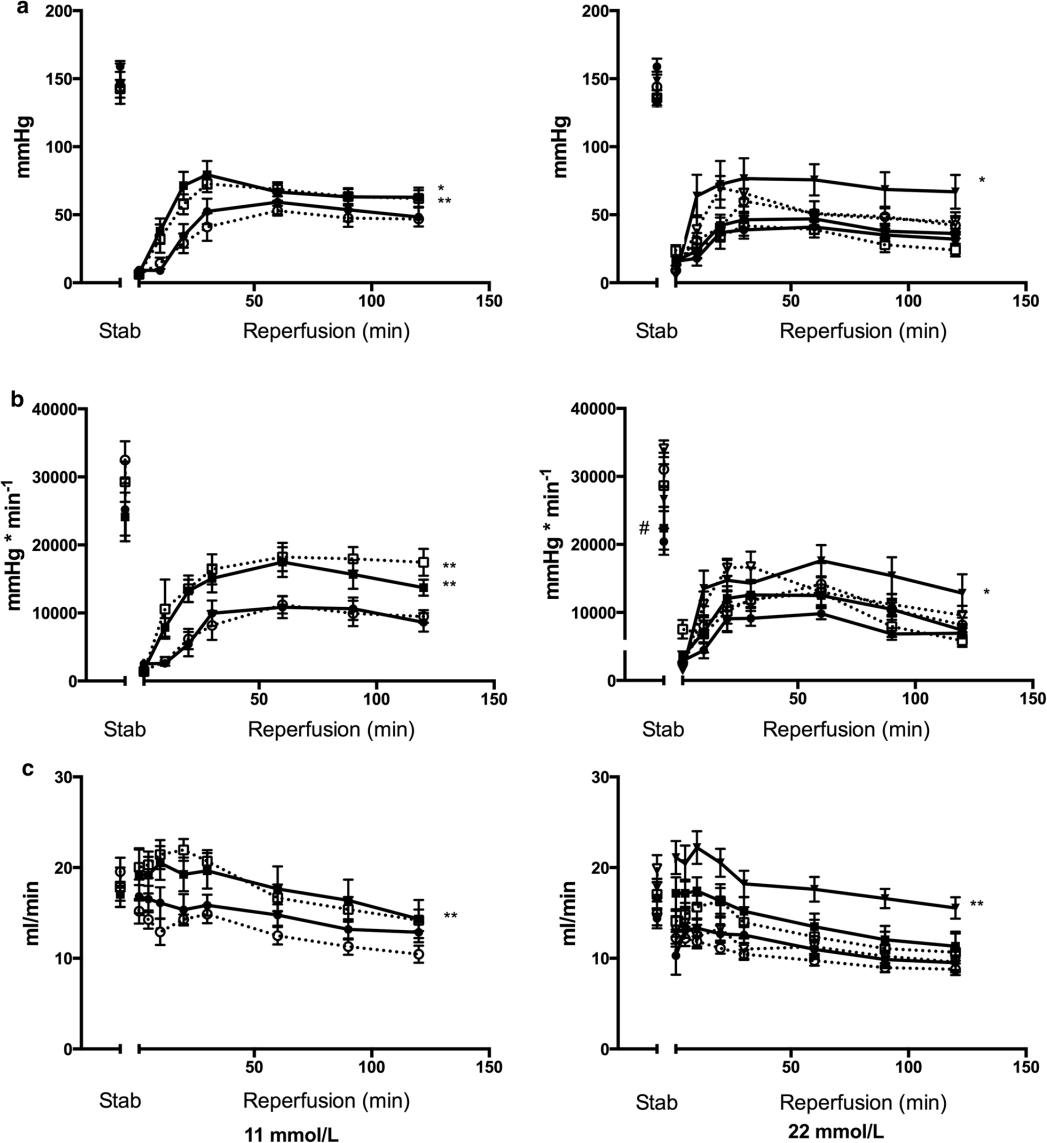
**a** Left ventricular developed pressure (LVDP), **b** rate pressure product and **c** coronary flow in diabetic (DM) Control (  ), DM ischemic preconditioned (IPC) (  ), DM intensified ischemic preconditioning (iIPC) (  ), Non-DM Control (  ), Non-DM IPC (  ) and Non-DM iIPC (  ) hearts during stabilization and reperfusion. Perfusion glucose level were 11 mmol/l and 22 mmol/L. ^#^p < 0.05 compared to non-DM Control. *p < 0.05; **p < 0.01 compared to control. Mean ± SEM

LVDP did not differ between control animals with and without T2DM during stabilization or reperfusion at either glucose level (Fig. 2a). LVDP did not differ between normo- and hyperglycemia in control animals with or without T2DM during stabilization or reperfusion. IPC increased LVDP during reperfusion in both animals with (p < 0.05) and without (p < 0.01) T2DM at normo- but not at hyperglycemia. Intensified-IPC increased LVDP during reperfusion in animals with (p < 0.05) but not without T2DM during hyperglycemia.

While RPP did not differ between animals with and without T2DM during stabilization at normoglycemia, RPP was decreased in animals with T2DM compared to animals without T2DM during stabilization at hyperglycemia (p < 0.05) (Fig. 2b). RPP did not differ between animals with and without T2DM during reperfusion at either glucose level. RPP did not differ between normo- and hyperglycemia in animals with or without T2DM during reperfusion. IPC increased RPP during reperfusion in animals with (p < 0.01) as well as without (p < 0.01) T2DM at normoglycemia but not at hyperglycemia. Intensified-IPC increased RPP during reperfusion in animals with (p < 0.05) but not without T2DM during hyperglycemia.

Coronary flow did not differ between animals with and without T2DM during stabilization (Fig. 2c) and was reduced at hyperglycemia in both animals with (p < 0.05) and without (p < 0.01) T2DM during reperfusion compared with normoglycemia. IPC increased coronary flow during reperfusion in animals without T2DM during normoglycemia (p < 0.01), whereas intensified IPC increased coronary flow in animals with T2DM during hyperglycemia (p < 0.01).

### Myocardial glucose uptake

**Fig. 3:**
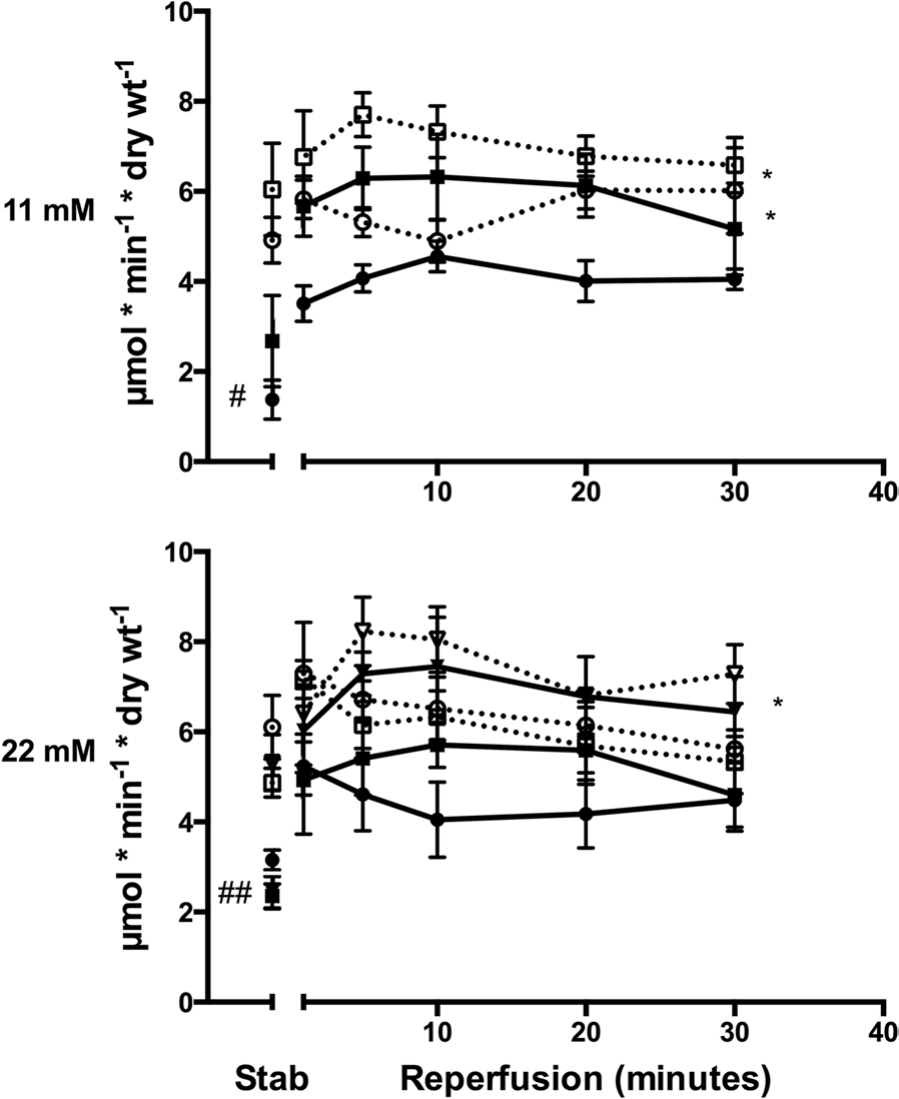
Tracer-estimated exogenous glucose uptake in diabetic (DM) Control (  ), DM ischemic preconditioned (IPC) (  ), DM intensified ischemic preconditioning (iIPC) (  ), Non-DM Control (  ), Non-DM IPC (  ) and Non-DM iIPC (  ) hearts during stabilization and reperfusion. Perfusion glucose level was 11 mmol/L and 22 mmol/L. ^#^p < 0.05; ^##^p < 0.01 compared to Non-DM Control. *p < 0.05 compared to control. Mean ± SEM

MGU was lower in animals with than without T2DM during hyper- and normoglycemia at both stabilization and reperfusion (Fig. 3). Hyperglycemia did not influence MGU in animals with and without T2DM during stabilization or reperfusion. While IPC increased MGU at normoglycemia in animals with (p < 0.05) and without (p < 0.05) T2DM during reperfusion, IPC did not change MGU in animals with or without T2DM at hyperglycemia. Intensified IPC increased MGU during reperfusion at hyperglycemia in animals with (p < 0.05) but not without T2DM.

### Myocardial O-GlcNAc concentrations

**Fig. 4:**
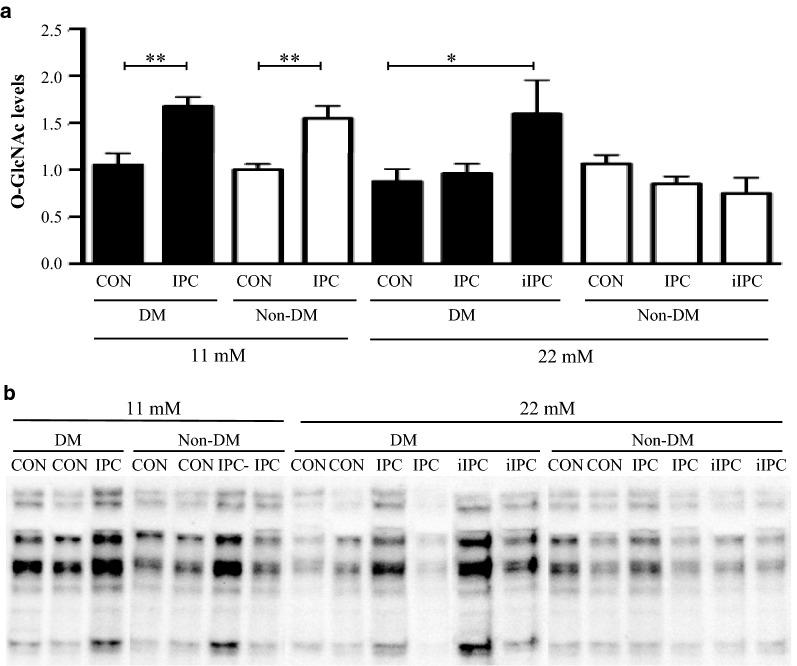
**a** O-GlcNAc (CTD110.6 antibody) levels in diabetic (DM) Control, Non-DM Control, DM ischemic preconditioned (IPC), Non-DM IPC, DM intensified ischemic preconditioned (iIPC) and Non-DM iIPC hearts expressed as fold change compared to 11 mmol/L Non-DM Control and correlated against total protein. **b** Representative O-GlcNAc bands. Note the higher intensity in the preconditioned compared with corresponding control hearts. *p < 0.05, **p < 0.01. Mean ± SEM

Myocardial levels of O-GlcNAc were similar in animals with and without T2DM during normo- and hyperglycemia (Fig. 4). Hyperglycemia induced no changes in O-GlcNAc levels compared with normoglycemia in animals with or without T2DM. IPC increased O-GlcNAc levels in animals with (p < 0.01) and without (p < 0.01) T2DM during normoglycemia but not during hyperglycemia. Intensified IPC increased myocardial O-GlcNAc levels in animals with (p < 0.05) but not without T2DM.

## Discussion

We demonstrate that hyperglycemia modifies endogenous and exogenous cardioprotection differently in rats with and without T2DM. In rats with T2DM, endogenous cardioprotection by T2DM is preserved during hyperglycemia, whereas the effect of exogenous cardioprotection by IPC is reduced and requires a stronger stimulus. In rats without T2DM, hyperglycemia abrogated exogenous cardioprotection also with an intensified stimulus. Myocardial glucose uptake rates and O-GlcNAc levels were only increased by exogenous cardioprotection by IPC and not by the endogenous cardioprotection observed in animals with recent onset T2DM. These findings suggest that different mechanisms of action are involved in exogenous cardioprotection induced by IPC and the endogenous cardioprotection observed in hearts from animals with T2DM.

### Myocardial susceptibility to ischemia–reperfusion injury

Circulating blood glucose levels influences the efficacy of endogenous cardioprotection. Here, we investigated specifically in hearts from animals with T2DM whether hyperglycemia had impact on endogenous cardioprotection. We find that infarct size in hearts from animals with T2DM is smaller than in hearts without T2DM during both normo- and hyperglycemia. This indicates that the endogenous cardioprotection observed in animals with T2DM during normoglycemia is preserved during hyperglycemia. We also investigated whether hyperglycemia per se influences myocardial susceptibility to ischemia–reperfusion injury. Myocardial infarct size was larger in animals with and without T2DM during hyperglycemia than during normoglycemia. This may indicate that myocardial susceptibility to ischemia–reperfusion injury is increased during hyperglycemia in T2DM to the same extend as demonstrated in animal models of type 1 diabetes [10, 11].

### Effects of ischemic preconditioning on IR injury

Circulating blood glucose levels also influences the efficacy of exogenous cardioprotection. We have recently reported that the effect of exogenous cardioprotection by IPC is preserved in animals with T2DM in contrast to animals without diabetes during hypoglycemia [9]. During hyperglycemia, a previous study failed to demonstrate an infarct sparring effect of preconditioning in hearts from animals with type 1 diabetes [11]. Here, we assessed the influence of hyperglycemia on the efficacy of exogenously induced cardioprotection in T2DM hearts and observed that IPC-induced infarct reduction was abrogated by a standard IPC protocol. However, using an intensified IPC protocol with four cycles of ischemia and reperfusion [7, 30], myocardial infarct size was reduced during hyperglycemia in hearts from animals with T2DM, while only to a minor and statistically insignificantly extend in hearts from animals without diabetes. These findings may reflect an additive effect of endogenous and exogenous cardioprotection in hearts from animals with T2DM and that hyperglycemia influences sensitivity to myocardial ischemia–reperfusion injury. The efficacy of IPC in hearts from animals with diabetes during normoglycemia remains controversial. The conflicting results may be related to the use of different models of diabetes. In models of T2DM, the cardioprotective effect of IPC during normoglycemia is abrogated [5, 31] or attenuated with an intensified preconditioning stimulus required to induce cardioprotection [7, 32, 33]. Here, we confirm that hearts from animals with T2DM are modifiable by exogenous cardioprotection by IPC during normoglycemia.

### Potential mechanisms

Evidence points towards alterations in myocardial glucose metabolism as a mechanistic link between preconditioning and cardioprotection because IPC is associated with increased glucose uptake during ischemia and reperfusion [12, 34–36]. We validated and extended the findings from normoglycemia to hyperglycemia by demonstrating that IPC-induced cardioprotection is associated with increased myocardial glucose uptake during hyperglycemia. Notably, the cardioprotective effect of exogenous cardioprotection by IPC is associated with increased myocardial glucose uptake in T2DM as well as non-diabetic hearts, while the endogenous cardioprotection observed in T2DM hearts is not. Hence, the underlying mechanisms of exogenously induced and endogenous cardioprotection seem to differ at a metabolic level. The IPC mediated increment in glucose uptake during ischemia–reperfusion has largely been attributed to activation of AMP-activated protein kinase (AMPK) and GLUT4 translocation [36]. Protein O-GlcNAcylation is sensitive to extracellular glucose levels and may also influence glucose uptake [37–39]. Exogenous cardioprotection by IPC and remote IPC involves modulation of myocardial O-GlcNAc levels and can be abrogated by azaserine, a commonly used inhibitor of glutamine:fructose-6-phosphate amidotransferase (GFAT), suggesting that O-GlcNAcylation is involved in the underlying mechanisms [15, 16, 28]. We extend these findings by demonstrating that exogenous cardioprotection by IPC during hyperglycemia is associated with increased myocardial levels of O-GlcNAc. In contrast, the endogenous cardioprotection observed in T2DM during both normo- and hyperglycemia was not associated with altered myocardial levels of O-GlcNAc suggesting different mechanisms of action between exogenous induced and endogenous cardioprotection at a post-translational level.

Alternative mechanisms of exogenously and endogenously induced cardioprotection may relate to the myocardial phosphatidylinositol 3-kinase/protein kinase B (PI3K/Akt) signaling system. The important role PI3/Akt signaling in ischemic preconditioning is well documented [40, 41], whereas it is reported to be defective in hearts from diabetic animals [7]. Furthermore, as hyperglycemia per-se suppresses Akt phosphorylation [42], our findings of reduced cardioprotection by IPC during hyperglycemia but preserved endogenous cardioprotection in diabetic animals are in support of a diverse role of PI3/Akt signaling in exogenously induced cardioprotection by IPC and the endogenous cardioprotection observed in hearts from diabetic animals. However, differences in PI3/Akt signaling do not seem to explain the disparate findings related to myocardial susceptibility to IR injury in different animal models of diabetes. In an animal models of type 1 diabetes mellitus induced by streptozotocin, decreased myocardial Sirt1 activity leading to decreased Akt phosphorylation and increased Drp1 activity increases myocardial susceptibility to IR injury [43]. In contrast, the Goto-Kakizaki rat, a lean model of T2DM, has lower myocardial levels of phosphorylated Akt and decreased myocardial susceptibility to IR injury. Future studies are needed to expand our understanding of the mechanisms related to differences in myocardial susceptibility to IR injury in different animal models of diabetes.

### Clinical implications

The clinical implications of our findings are that myocardial infarct size in patients with or without T2DM may be influenced by changes in myocardial susceptibility to ischemia–reperfusion injury caused by hyperglycemia per-se similarly to the animals investigated in the present study. Furthermore, the effect of exogenous cardioprotection activated prior to an ACS may partly or completely be abrogated by hyperglycemia in patients with or without T2DM, which may result in increased myocardial infarct size. As myocardial infarct size is a prognostic factor after acute myocardial infarction, this finding may in part explain the observed increased mortality in patients with ACS and elevated circulating glucose levels at hospital admission. However, as infarct sizes at both normo- and hyperglycemia were smaller in animals with than without T2DM, infarct size alone does not seem to explain the overall higher mortality in patients with T2DM suffering acute myocardial infarction compared with patients without diabetes. The higher mortality in patients with diabetes may merely be related to arrhythmias after acute myocardial infarction, as suggested by findings of exogenously induced conditioning being ineffective against IR-induced atrioventricular block in animals with diabetes in contrast to animals without diabetes [44].

### Limitations

A strength of the present study is the use of a clinically relevant animal model of T2DM as patients with T2DM prevail in clinical patient cohorts of acute myocardial infarction. Yet, our T2DM rats were rather young with recent onset of diabetes and the concurrent effect of age was not investigated although T2DM is often diagnosed in elderly patients. A limitation of the study relates to the use of an isolated perfused heart model. However, induction of hyperglycemia in vivo influences insulin production and levels, which impacts myocardial ischemia–reperfusion injury [45] and may influence myocardial glucose uptake and O-GlcNAc levels differently in T2DM and non-diabetic hearts. Furthermore, the influence of the systemic response to hyperglycemia including activation of neurogenic and humoral components can be excluded in our model. Our diabetic animal were exposed to hyperinsulinemia until the time of IR, which was performed without insulin as this per se influences cardioprotection [46]. Performing IR during hyperinsulinemia in diabetic animals and during normoinsulinemia in non-diabetic animals in our model is not believed to have impacted the comparison of the results between animals with and without diabetes substantially as the mechanism whereby insulin leads to Akt activation is impaired in the diabetic state by upregulation of phosphatase and tensin homolog deleted on chromosome 10 (PTEN) [47, 48]. However, as hyperglycemia diminishes the cardioprotective effects of insulin [42], the presence of insulin during IR may have augmented the difference in IR injury between normo- and hyperglycemia in animals without diabetes. As the ZDF model of T2DM is also characterized by high circulating cholesterol, triglycerides and free fatty acids concentrations [5], our diabetic animals were also exposed to hyperlipidemia until the time of IR. Conflicting data regarding the impact of hyperlipidemia on cardioprotection have been reported [49, 50]. However, the potential influence of hyperlipidemia on cardioprotection does not require presence of the lipids at the time of IR as hyperlipidemia prior to IR has been demonstrated to modulate cardioprotection in isolated hearts perfused with Krebs–Henseleit buffer containing glucose as the only substrate in the absence of lipids [51, 52]. Such findings may be explained by altered myocardial signaling, gene and microRNA profiles caused by exposure to hyperlipidemia [50] and such effects would also present in our study. We specifically chose to investigate the effects of hyperglycemia on myocardial infarct size in a glucose-dependent model to investigate the impact on MGU without interference from other substrates including lipids and ketones. We cannot exclude that our findings may have been influenced by substrate competition in the presence of free fatty acids as well as other substrates. Variations in circulating glucose concentrations changes osmolarity, which may influence infarct size and an increase in plasma glucose from 11 to 22 mmol/L leads to a 3% increase in osmolarity of the perfusion buffer. The influence of osmolarity on infarct size has varied in previous studies. Increases in serum osmolarity by administration of raffinose did not modify infarct size or impact the capacity of IPC to protect against infarction as demonstrated by Kersten et al. [11]. In contrast, Zalesak et al. demonstrated that a hyperosmotic state reduced the cardioprotective capacity of IPC and decreased myocardial susceptibility to ischemia–reperfusion injury suggesting that increased osmolarity may play a role in lack of cardioprotection by IPC in the myocardium from animals with diabetes [23]. In the present study, glycemic and osmolarity levels were altered similarly by hyperglycemia and as infarct size was similarly in both animals with and without diabetes during hyper-compared with normoglycemia we conclude that the increased osmolarity during hyperglycemia in the present study does not influence myocardial ischemia–reperfusion injury. We cannot establish whether the blunted effect of IPC, although still present with an intensified stimulus in animals with T2DM, was caused by hyperglycemia or an increase in osmolarity.

## Conclusion

Hyperglycemia does not abrogate the endogenous cardioprotection in hearts from animals with recent onset T2DM. The efficacy of exogenous cardioprotection by IPC is reduced during hyperglycemia but preserved when an intensified stimulus is used in animals with T2DM. The underlying mechanisms of exogenous cardioprotection by IPC seem to involve increased myocardial glucose uptake and O-GlcNAc levels and the effects of endogenous and exogenous cardioprotection by IPC seem to be additive in T2DM.

## Data Availability

Not applicable. The conclusions of the manuscript are based on relevant data sets available in the manuscript.

## Abbreviations

AAR: area at risk
ACS: acute coronary syndrome
ANOVA: analysis of variance
DM: diabetes mellitus
HBP: hexosamine biosynthetic pathway
IPC: ischemic preconditioning
IR: ischemia–reperfusion
IS: infarct size
LVDP: left ventricular developing pressure
MGU: myocardial glucose uptake
O-GlcNAc: O-linked β-*N*-acetylglucosamine
RPP: rate pressure product
T2DM: type 2 diabetes mellitus
ZDF: Zucker diabetic fatty
